# Create Guidelines for Characterization of Venom Peptides

**DOI:** 10.3390/toxins8090252

**Published:** 2016-08-30

**Authors:** Volker Herzig

**Affiliations:** Institute for Molecular Bioscience, The University of Queensland, St. Lucia, QLD 4072, Australia; v.herzig@imb.uq.edu.au; Tel.: +61-7-3346-2018; Fax: +61-7-3346-2021

In the course of my duties as a curator for the ArachnoServer database [1,2], I recently came across the article published by Binda et al. in *Toxins* [3]. In this article, the effects of a toxin termed **PhTx3-4** (isolated from the venom of the Brazilian wandering spider *Phoneutria nigriventer*) are studied in an animal model of retinal injury, in which the toxin showed neuroprotective effects from NMDA-induced retinal injury. According to this article, PhTx3-4 has a native molecular mass of 8449 Da. However, the sequence that is provided has a predicted (average oxidised) mass of 8459 Da, which is a difference of 10 Da. Given the accuracy of mass spectrometry, this difference cannot simply be explained by an imprecise measurement. For the suggested activity of PhTx3-4 on voltage-gated calcium channels, the authors then referred to the sequence of ω-Phonetoxin IIA (**ω-PtxIIA**) as published by dos Santos et al. [4]. However, the sequence of ω-PtxIIA has six residues difference to PhTx3-4 and the molecular mass of ω-PtxIIA is 8363 Da, which is 86 Da less than the native mass of PhTx3-4. Furthermore, there is also a difference in the cysteine-framework in position 15 and 19 of these toxins, which could have a significant impact on the toxin folding and activity (Figure 1).

Further confusion in regards to the PhTx3-4 sequence is then created by referring to the NCBI accession number P81790 (=ω-ctenitoxin-Pn3a or ω-CNTX-Pn3a), which has originally been described as **PNTx3-4** [5]. A closer look at the sequence shows that PNTx3-4 is five residues different to PhTx3-4 (including the above-mentioned difference in the cysteine-framework), with one additional C-terminal glycine in comparison to ω-PtxIIA. PNTx3-4 has a molecular mass of 8420 Da, which is 29 Da less than the molecular mass of the native PhTx3-4. Thus, PhTx3-4, ω-PtxIIA, and PNTx3-4 are actually three different toxins, and (as indicated in the alignment in Figure 1) another homologous toxin sequence called **Pn3-4A** has also been described [6].

Despite these obvious differences in the toxin sequences (Figure 1) and masses (Table 1), the authors of the Binda et al. article mix up these toxins when referring to the activity and sequence of PhTx3-4, which was actually the only toxin used in their experiments [3]. However, just because a toxin was isolated from venom of the same species and has similar activity as an already-characterized toxin from that venom, it does not imply that both toxins are identical (i.e., have the same mature toxin sequence). It is well known that spiders often produce many homologs of a single toxin to increase their chemical diversity of venom peptides [10]. Vice versa, if two homologous toxins have slight differences in their toxin sequence, one cannot simply conclude that these toxins will have a similar activity at a certain target, especially if the cysteine framework is not identical. In some cases, a single residue difference or even a C-terminal amidation can significantly alter the activity of a toxin [11].

The first 40 residues of the sequence of PhTx3-4 presented by Binda et al. actually match the N-terminal sequence reported for **Tx3-4** [8]. Given than the native molecular masses of these toxins also match, I would assume that at least one of the residues in position 41–77 in the complete sequence provided for PhTx3-4 was not correctly assigned (as indicated in Figure 1), which would explain the 10 Da difference in molecular mass between the native toxin and the proposed sequence (the respective toxin card in ArachnoServer now also reflects this discrepancy). Hence, a digestion of the toxin followed by N-terminal Edman sequencing of the unclear C-terminal residues is required to clarify this issue and to once and for all provide the correct and complete sequence of PhTx3-4.

The fact that the correct sequence of PhTx3-4 (matching the native molecular mass of 8449 Da) still remains unknown makes it impossible for other researchers (which do not have access to the crude venom of *P. nigriventer*) to verify the results presented by Binda et al. [3] (e.g., by using chemically synthesized or recombinantly produced toxin). However, it is an essential part of science to be able to reproduce results from other researchers in order to verify their results. Thus, all journals that publish scientific studies have to ensure that all articles they publish provide sufficient detail and correct data to ensure that experiments can be verified.

For future publications in *Toxins* (although this also applies to other journals that publish articles on peptide toxins), I would therefore suggest the establishment of guidelines that must be met by authors before their results on peptide toxins can be published. These guidelines must be defined in a way that will allow another researcher in a different lab to repeat the experiment(s). These guidelines should, for example, include:

1. **Details on the type of venomous animal** (e.g., correct taxonomic identification of the species, including a statement on the expert identification or the respective references used for identification) from which the venom was sourced. The geographical origin, which is known to affect venom composition, should also be indicated as precisely as possible. However, this might sometimes be challenging if the specimen was purchased from commercial suppliers, who are not always willing to share this information. In case there is no information available about the geographical origin, this should be stated explicitly. Ideally, a voucher specimen should be deposited within a museum collection to enable a later verification of the correct taxonomic identification and determination of possible phylogenetic relationships, although this might not always be a feasible option. The method that was used for venom extraction (e.g., electrical stimulation or dissection of the venom gland) or details of the venom supplier should also be indicated.
2. **The method used to isolate the toxin** (e.g., HPLC instrument, gradient, solvents, flow rates, detection wavelength, column type, size, and material, etc.) and the results of the toxin isolation (e.g., a figure of the respective HPLC traces, ideally indicating the gradient and highlighting the active peak(s)).
3. **Mass spectrometry data** (including all details on the type of MS that was used) confirming the molecular mass and purity of the native toxin.
4. **The method used for sequence determination** of the toxin (N-terminal Edman degradation or MS-MS, types of instruments used, detailed protocols for each of these sequencing methods) and the results of the sequencing. Ideally, to facilitate a thorough check of the sequence, the full peptide sequence should be included in the text body of the manuscript and not only in figures (from where it can't be simply copied and pasted, e.g., onto other websites).
5. **The native and predicted toxin mass need to match.** Thus, the authors need to provide a proper discussion showing that the mass of the native toxin is within 1 Da of the molecular mass predicted from the sequencing results, unless the authors can provide convincing evidence for larger differences (e.g., in case of post-translational modifications).
6. **Each toxin needs a unique name.** In order to avoid confusion with other toxins with the same name, the authors need to ensure that the name that they choose has not been used before for any other peptide toxin. Ideally, the naming of mature peptide toxins should follow the rational nomenclature for peptide toxins [7], which is recommended by the International Society on Toxinology. For any spider venom peptide that has not been named according to the rational nomenclature for peptide toxins, the curators of ArachnoServer will assign the recommended name and keep the previous name as a synonym. The names recommended by ArachnoServer are then automatically updated in UniProt.

These points listed here might not be complete, but should provide a basis for discussion on the minimum standards that an article has to meet to be considered for publication. Using these guidelines will ensure a high standard for scientific publications and help all scientists to obtain all the information from published literature that is essential for their research.

## Figures and Tables

**Figure 1:**
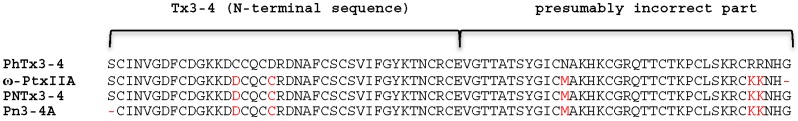
Sequence alignment of toxins from the ω-ctenitoxin-Pn3 family with close sequence homology to PhTx3-4 (extracted from ArachnoServer database on 17.3.2016). All residues that are different to the sequence proposed for PhTx3-4 [3] are in red colour.

**Table 1:** **The ω-ctenitoxin-Pn3 toxin family.** Overview of the four homologous toxins listed in Figure 1, with details on their ArachnoServer accession number (AS#), the recommended toxin name (based on the rational nomenclature for peptide toxins [7]), the toxin synonym, the average oxidised mass in Da, and the original reference (all data extracted from ArachnoServer database on 17.3.2016).

AS#	Toxin name	Synonym	Mass (Da)	Reference
2365	ω-ctenitoxin-Pn3d	PhTx3-4	8449 (native)	[3,8]
2364	ω-ctenitoxin-Pn3c	ω-PtxIIA	8362.6	[9]
262	ω-ctenitoxin-Pn3a	PNTx3-4	8419.6	[5]
457	ω-ctenitoxin-Pn3b	Pn3-4A	8332.5	[6]
